# The global prevalence of tooth wear in general population: a systematic review and meta-analysis

**DOI:** 10.1016/j.puhip.2025.100708

**Published:** 2025-12-12

**Authors:** Nader Salari, Amir Hossein Sadeghi, Amir Abdolmaleki, Hosna Zarei, Amir Hossein Ghaderi, Shamarina Shohaimi, Masoud Mohammadi

**Affiliations:** a Department of Biostatistics, School of Health, Kermanshah University of Medical Sciences, Kermanshah, Iran; b Student Research Committee, Kermanshah University of Medical Sciences, Kermanshah, Iran; c Department of Operating Room, Nahavand School of Allied Medical Sciences, Hamadan University of Medical Sciences, Hamadan, Iran; d Medical Biology Research Centre, Kermanshah University of Medical Sciences, Kermanshah, Iran; e Department of Biology, Faculty of Science, University Putra Malaysia, Serdang, Selangor, Malaysia; f Research Center for Social Determinants of Health, Jahrom University of Medical Sciences, Jahrom, Iran

**Keywords:** Prevalence, Tooth wear, Caries, Erosion, Attrition

## Abstract

**Objective:**

Tooth wear (TW), as a common dental challenge, refers to the loss of dental tissue with four types of Attrition, Erosion, Abrasion, and Abfraction. Since there are no scientific reports regarding the global TW prevalence, this study was designed to investigate the prevalence of TW worldwide.

**Study design:**

Systematic review and meta-analysis.

**Methods:**

Systematic search was conducted (by November 2024) in valid databases of PubMed, WoS, ScienceDirect, Scopus, Embase, and Google Scholar search engine using the main keywords of “Prevalence”, “Tooth wear”, “Erosion”, “Attrition”, “Abfraction”, and “Abrasion”. The I^2^ index was used to examine heterogeneity, and the Random Effect Model was used for meta-analysis (CMA v.2).

**Results:**

Following the review of 133 eligible studies with the sample size of 92,153 individuals with TW, the global prevalence of TW was found to be 40.8 % (95 %CI: 36.7–45, I^2^: 99.1).

**Conclusion:**

According to the high global prevalence of TW, the implementation of a comprehensive strategy by the health care system seems necessary to control TW occurrence and prevent the associated complications.

## 1 Introduction

Tooth wear (TW) is a common dental health problem with a low rate of public awareness in this regard. Although certain superficial changes in dental hard tissues may be considered part of normal physiological processes [[1], [2], [3]], scientifically, the TW occurs due to the demineralization caused by oral fermentation of carbohydrates [5]. TW is a broad dental term which encompasses various forms of non-carious loss of dental hard tissue with four main subclassifications according to distinct underlying mechanisms, including Erosion, Attrition, Abrasion, and Abfraction [6,7].

TW is a clinically observable phenomenon which often poses significant diagnostic and therapeutic challenges, requiring comprehensive assessment and tailored management strategies to address the associated multifactorial etiology and prevent further deterioration. Historical and contemporary epidemiological studies indicate that early signs of tooth attrition are observed in the vast majority of adults [[3], [4], [5], [6], [7]], but there are also more severe forms which probably affect approximately 10 % of the population. Identification of the underlying cause, determination of the optimal timing to initiate preventive measures, and precise decision-making regarding the necessity for surgical intervention are essential components for effectively planning the long-term management of patients with tooth attrition. These steps ensure tailored care which addresses disease progression and preserves dental function [[3], [4], [5], [6], [7]].

Attrition usually occurs following direct contact between the teeth. Erosion is the most common type of TW following chemical exposure, including carbonated drinks, and gastroesophageal reflux disease (GERD). Enamel destruction is seen widely in erosion. Abrasion is detected following hard object collision with serious damage to the gingiva and tooth tissue, such as improper toothbrushing, contact with orthodontic prostheses, and nail biting. In abfraction, the cervical surface of teeth is damaged, particularly at the junction between hard enamel and softer cementum [7].

Various studies report differing prevalence rates of TW across countries and age groups, including 1–34 % in 3–6 years, 21–81 % in 6–9 years, and 1–53 % in 12–14 years children [10,11]. Also, the prevalence of TW was found to be 28 %, 34.1 %, 60.2 %, and 58 % in Turkey [12], Brazil [13], the UAE, and Colombia [14], respectively, in the general population [15]. According to recent studies, no specific factor exists regarding the occurrence of TW in children, while some other studies reported the detrimental role of GERD and carbonated drinks on TW. Smoking is another important factor in TW occurrence, along with many demographic indicators (such as age and educational level) [16], medications, and teeth grinding [17]. The presence of multiple influencing factors on TW occurrence creates difficulties in the identification of the main cause of TW. In this regard, the diagnosis of dental damage faces many challenges [18].

According to the different reports of TW prevalence, the authors of the present study aimed to investigate the global prevalence of TW along with associated influencing factors.

## 2 Methods

All protocols of the present systematic review and meta-analysis were followed by PRISMA guidelines. Inclusion criteria were all cross-sectional studies reporting the prevalence of TW. All included papers were prepared in both Persian and English languages with available full text and extractable data. Also, exclusion criteria were experimental studies, articles with no available full text, and duplicate investigations. According to the PICO index, the study population (P) included general population, and the outcome (O) included the TW prevalence. Also, no intervention (I) and comparison (C) exist for the current study.

Main keywords were selected based on the MeSH concept, including “Prevalence”, “Tooth wear”, “Erosion”, “Attrition”, “Abfraction”, and “Abrasion”. The searching process was conducted in valid databases of PubMed, Embase, WoS, Scopus, ScienceDirect, and the Google Scholar search engine. Also, the references of selected papers were reviewed to achieve the maximum number of eligible articles. Initial searching was conducted on July 21, 2024, and data was updated on November 26, 2024.

### 2.1 Study selection

Studies were selected according to the PRISMA guidelines. Initially, duplicate studies were excluded (using Citation Management Software of EndNote). Then, the remaining studies were enrolled by primary and secondary screenings. In this regard, the Title and Abstract of studies were assessed (primary screening) according to the inclusion and exclusion criteria. Then, the full text of the papers was reviewed regarding the data extractability (secondary screening). All irrelevant studies were excluded, and the remaining eligible investigations underwent quality control assessment. Study selection was performed by two researchers, and the corresponding author was responsible for management of any disagreements in findings.

### 2.2 Qualitative assessment

The Strengthening The Report of Observational Studies in Epidemiology (STROBE) checklist was used to assess the quality of studies. The main parts of the included articles were assessed for quality control, including Title, Abstract, Introduction, Methods, Results, and Discussion. Also, subcategories including Study objective, Problem statement, Study type, Statistical population, Sampling method, Determination of sample size, Definition of variables, Data collection tools, Statistical analysis methods, and Findings were extracted and recorded. Finally, STROBE scores <16 were considered poor-quality and excluded; besides, STROBE scores≥ 16 were high-quality investigations and included for data extraction.

### 2.3 Data extraction and meta-analysis

Data extraction was applied using a previously prepared checklist, including the Name of the first author, Year of paper publication, Country, Study type, Number of samples, Number of individuals with TW, Percentage of individuals with TW, Age range, Data collection tools, and Type of TW. The comprehensive meta-analysis software (CMA, v2) was used for data analysis. I^2^ and Egger tests were used for heterogeneity and publication bias assessments, respectively.

## 3 Results

**Fig. 1:**
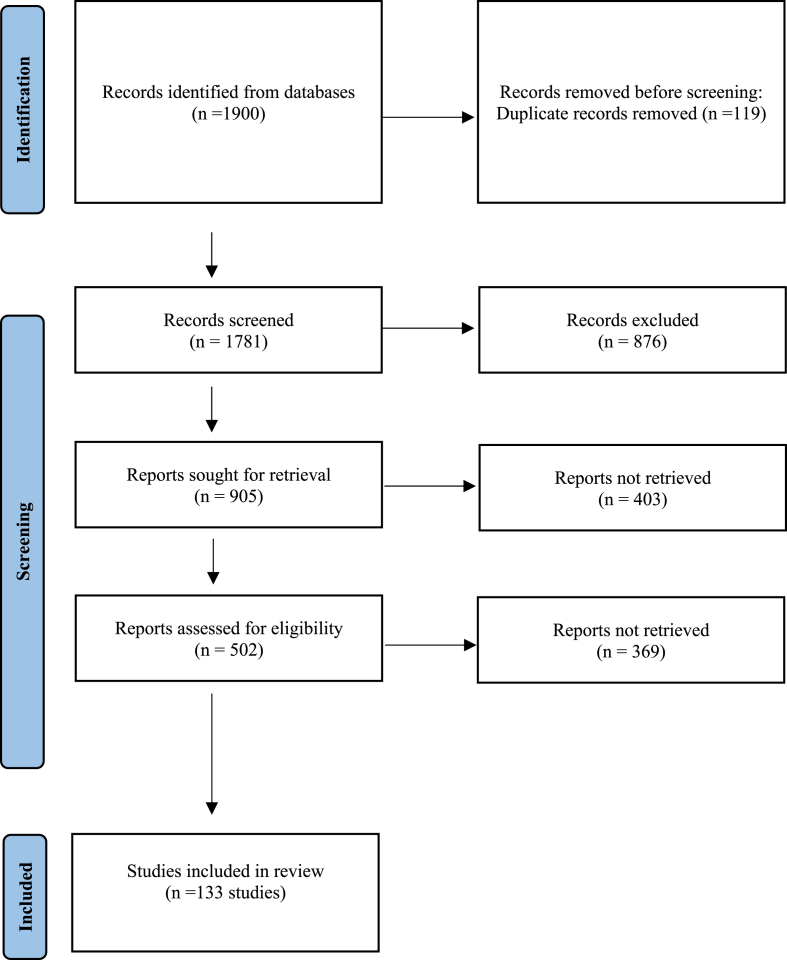
RISMA flow diagram representing the process of relevant study identification.

During systematic searching, 1900 studies (PubMed:239, Google scholar:820, Scopus:187, Embase:86, Web of Science:245, and ScienceDirect:323) were found. Using EndNote software, 119 and 876 duplicate and irrelevant articles were excluded, respectively. Also, 403 investigations were removed according to the inclusion and exclusion criteria. In the qualitative assessment, 369 low-quality investigations were also excluded. Finally, 133 high-quality studies were included for data extraction (Fig. 1 and Table 1).

**Table 1:** Study characteristics of included studies reporting the prevalence of tooth wear.

First author	Year	Country	Sample size	Prevalence	Age	Instrument	Type of tooth wear	Qualitative assessment
Noorhazayti Ab Halim et al. [3]	2018	Malaysia	598	45	16	Questionnaire	Erosion	High
S.B.AbuGhazaleh et al. [6]	2013	Jordan	1602	51	15–16	Clinicalexamination and questionnaire	General	Moderate
Ary.Agustanti et al. [5]	2018	Indonesia	487	63	12	Clinicalexamination and questionnaire	General	High
Manal Al Halabi et al. [11]	2016	UAE	506	97.6	2–8	Clinical examination and questionnaire	97.6	Moderate
M. Al-Malik et al. [19]	2000	Saudi arabia	80	30	4–5	Clinicalexamination	Erosion	Moderate
Al-Hammadi S et al. [7]	2020	Yemen	600	87.3	20–50	Clinicalexamination and questionnaire	General	High
Al-Khalifa, K. S. et al. [20]	2020	Saudi arabia	340	83.5	18–40	Questionnaire	General	High
Al-Majed I et al. [21]	2002	Saudi arabia	1216	28.28	5-6-, 12-14	Clinical examination and questionnaire	Erosion	High
Alvarez Loureiro L et al. [22]	2015	Uruguay	1136	52.9	12	Clinicalexamination and questionnaire	Erosion	Moderate
Arnadottir I. B et al. [18]	2010	Iceland	2251	46.4	15, 12, 6	Clinical examination and questionnaire	Erosion	Moderate
Auad Sheyla M et al. [13]	2007	Brazil	458	34.1	13–14	Clinicalexamination and questionnaire	Erosion	Moderate
Avila VBetlrán E.et al. [15]	2023	Colombia	454	71.6	12–15	Clinicalexamination and questionnaire	Erosion	High
Britton Karen, F. M. et al. [23]	2009	Scotland	188	37.2	6m-6y	Clinicalexamination	Erosion	Moderate
Brusius C. D et al. [24]	2018	Brazil	801	10	2–5	Clinicalexamination	Erosion	High
Buczkowska-Radlińska J et al. [25]	2013	Poland	62	26	14–16	Clinicalexamination and questionnaire	26	Moderate
Caglar E et al. [26]	2007	Turkey	153	28	11	Questionnaire	Erosion	Moderate
Caglar E et al. [27]	2011	Turkey	83	45.8	7–14	Clinicalexamination and questionnaire	Erosion	Moderate
Caraguay Martínez Johana et al. [28]	2018	Ecuador	175	53.4	8–12	Clinicalexamination	General	Moderate
Caye Luís Felipe Sá et al. [29]	2020	Brazil	116	21.6	–	Clinical examination and questionnaire	Erosion	Moderate
Chavan Jyothi et al. [30]	2017	India	175	80	25–34	Clinicalexamination	Erosion	High
Christopher Deery B et al. [31]	2000	USA ,UK	254	38.97	11–13	Clinicalexamination	Erosion	Moderate
Chrysanthakopoulos, N. A et al. [32]	2012	Greece	770	33.76	13–16	Clinicalexamination and questionnaire	Erosion	Moderate
Corra Nahs et al. [33]	2011	Brazil	232	25.43	2–20	Clinicalexamination and questionnaire	Erosion	Moderate
Cunha-Cruz Joana et al. [34]	2008	USA	1288	51	18–93	Clinicalexamination	General	Moderate
de Carvalho Sales-Peres S. H,et al. [35]	2008	Brazil	295	26.9	12	Clinicalexamination	General	High
Deshpande S. D et al. [36]	2004	India	100	30	5–6	Clinicalexamination	Erosion	Moderate
Dimitrova M et al. [37]	2021	Bulgaria	222	57.67	3–7	Clinicalexamination and questionnaire	General	High
Donachie M. A et al. [38]	1995	UK	1002	58.6	>45	Clinicalexamination	General	Moderate
Duangthip D et al. [39]	2018	Hong kong	1204	14.9	3–5	Clinicalexamination and questionnaire	Erosion	Moderate
Dugmore C. R, Rock W. P. et al. [40]	2004	UK	1753	59.7	12	Clinicalexamination	Erosion	Moderate
Tarsitsa Gatou et al. [41]	2012	Greece	243	51.8	5–7	Clinical examination and questionnaire	General	High
Gennadievna M. N et al. [42]	2022	Russia	98	57.2	17–64	Clinicalexamination	Abrasion	Moderate
Farahat Farnaz et al. [43]	2016	Iran	400	21.1	>12	Clinical examination and questionnaire	Erosion	Moderate
Frazao J. B et al. [44]	2018	Brazil	239	11.7	6–10	Clinicalexamination and questionnaire	Erosion	Moderate
Gillborg S et al. [45]	2020	Sweden	831	80	20–89	Clinicalexamination and questionnaire	General	Moderate
González-AragónPineda ÁlvaroEdgar,et al. [46]	2016	Mexic	417	31.7	14–19	Clinicalexamination and questionnaire	Erosion	Moderate
Gopinath, V. K. et al. [47]	2016	UAE	403	58.8	5	Clinicalexamination	Erosion	Moderate
Goyal Kriti et al. [48]	2023	India	206	39.3	>18	Questionnaire	General	High
Graves R. C et al. [49]	1992	USA	809	24.9	>65	Clinical examination and questionnaire	General	Moderate
Gurgel C. V et al. [50]	2011	Brazil	414	20	12–16	Clinicalexamination and questionnaire	Erosion	High
Habib M et al. [51]	2013	USA	243	13	2–4	Clinicalexamination and questionnaire	Erosion	Moderate
Hanoon, S. A. et al. [52]	2021	Iraq	1000	10.2	11–12	Questionnaire	Erosion	Moderate
Harłukowicz K et al. [53]	2017	Poland	240	16.25	12–18	Clinical examination and questionnaire	Erosion	High
Hegde Mithra N et al. [54]	2018	India	1000	40.6	40–60	Questionnaire	General	High
A. L-Sultani HF et al. [55]	2013	Iraq	100	74	20–40	Clinicalexamination	General	Moderate
Hou X. M et al. [56]	2009	China	864	61.8	12	Questionnaire	Erosion	Moderate
Huew R et al. [57]	2012	Libya	791	40.8	12	Clinical examination and questionnaire	Erosion	Moderate
Hugoson A et al. [58]	1988	Sweden	585	76	20–80	Clinicalexamination	General	Moderate
Hugoson A et al. [59]	1996	Sweden	527	50.8	3–20	Clinicalexamination and questionnaire	General	Moderate
Imran M et al. [60]	2020	Pakistan	310	9.5	21–65	Clinicalexamination	Attrition	Moderate
Ismail Rebaz Samir et al. [61]	2024	Iraq	176	93	40	Clinicalexamination	Attrition	High
Jaeggi T et al. [62]	2004	Switzerland	42	47.6	5–9	Clinicalexamination	Erosion	Moderate
Urszula Kaczmarek et al. [63]	2012	poland	181	16.6	15	Clinicalexamination	Erosion	Moderate
Jász, Máté et al. [64]	2022	hungray	609	22	12	Questionnaire	Erosion	High
Kan S et al. [65]	2017	China	1812	57.16	12–74	Clinicalexamination and questionnaire	General	Moderate
Kaptan A et al. [66]	2020	Turkey	473	21.18	7–14	Clinicalexamination	Erosion	High
Kirthiga M et al. [67]	2015	India	2000	1.4	11–16	Clinicalexamination and questionnaire	Erosion	Moderate
Kitasako Y et al. [68]	2017	Japan	1108	26.1	15–89	Clinical examination and questionnaire	Erosion	High
Khan G. A et al. [69]	2022	Pakistan	1021	12.2	3–5	Clinicalexamination	Erosion	High
H.*M. van* Rijkom et al. [70]	2002	The Netherlands	745	17.45	10-13, 15-16	Clinicalexamination	General	Moderate
Korkmaz E et al. [71]	2020	Turkey	473	21.8	7–14	Questionnaire	Erosion	High
Kumar S et al. [72]	2013	India	605	8.9	11–14	Questionnaire	Erosion	Moderate
Li JianBo et al. [73]	2019	China	720	56.1	15–55	Questionnaire	Erosion	Moderate
Lara Icay et al. [74]	2022	Uruguay	614	73.1	5	Clinical examination and questionnaire	Erosion	High
Larsen M. J et al. [75]	2005	Denmark	558	13	15–17	Clinicalexamination	Erosion	Moderate
Liu B et al. [76]	2014	China	704	88.16	–	Clinicalexamination and questionnaire	General	High
Longbottom, Chris et al. [77]	2004	UK	1573	59.17	12	Clinicalexamination	Erosion	Moderate
Luciano L. C. O et al. [78]	2017	Brazil	335	86.5	12–30	Clinical examination and questionnaire	Erosion	High
Luo Y et al. [79]	2005	China	1949	5.7	3–5	Clinicalexamination	Erosion	Moderate
Lussi A et al. [80]	1991	Switzerland	391	81	20-30, 46-50	Clinicalexamination	Erosion	Moderate
Al-Hiyasat A et al. [81]	2006	Jordan	143	90.9	26–35	Clinicalexamination and questionnaire	General	Moderate
Mafla A. C et al. [82]	2017	Colombia	384	57.3	10–15	Clinicalexamination	Erosion	High
Maharani D. A et al. [83]	2019	Indonesia	691	23	5	Clinicalexamination and questionnaire	General	High
Marqués Martínez L et al. [84]	2020	Spain	391	19.7	5–12	Clinicalexamination	Erosion	High
Marró M et al. [85]	2020	Germany	535	97.9	18–46	Clinicalexamination	Erosion	High
Minh Son T et al. [86]	2018	Vietnam	800	88.4	18–60	Clinicalexamination	General	Moderate
Methuen M et al. [87]	2022	Finland	325	36.9	>15	Clinical examination and questionnaire	Erosion	High
Millward A et al. [88]	1994	Canada	178	50	4	Clinicalexamination	Erosion	Moderate
Milosevic A et al. [89]	1994	UK	1035	30	14	Clinicalexamination	General	Moderate
Mirani Shahid, Ali et al. [90]	2014	Pakistan	200	32	18–25	Questionnaire	Erosion	High
Mohamed R et al. [91]	2021	Saudi arabia	1200	27.6	6–16	Clinicalexamination and questionnaire	Erosion	High
Moimaz S. A et al. [92]	2013	Brazil	2759	0.6	4–6	Questionnaire	Erosion	Moderate
Mulic A et al. [93]	2016	Norway	392	38	16	Clinicalexamination	Erosion	High
Mulic A et al. [94]	2011	Norway	48	31.25	–	Clinicalexamination and questionnaire	Erosion	Moderate
Mulic A et al. [95]	2013	Norway	1456	18.3	18	Clinicalexamination	Erosion	Moderate
Muller-Bolla M et al. [96]	2015	France	336	39	14	Questionnaire	Erosion	Moderate
Mungia R et al. [97]	2009	USA	307	5.5	12–18	Questionnaire	Erosion	Moderate
Murakami C et al. [98]	2016	Brazil	2801	51.6	3–4	Clinicalexamination	Erosion	Moderate
Mužinić K et al. [99]	2019	Croatia	25	48	11–16	Clinicalexamination and questionnaire	Erosion	High
Nicholas Lim et al. [100]	2021	Singapore	1296	21.8	18–25	Clinicalexamination and questionnaire	Erosion	High
Nahás Pires Corrêa M. S et al. [101]	2011	Brazil	232	25.43	2–20	Clinicalexamination	Erosion	Moderate
Nakane Ayako et al. [102]	2014	Japan	116	34	2–6	Clinicalexamination and questionnaire	Erosion	High
Nayak S. S et al. [103]	2009	India	200	22	12	Questionnaire	Erosion	Moderate
Nayak S. S et al. [104]	2010	India	1002	29	5	Clinicalexamination and questionnaire	Erosion	High
Noh Taehwan et al. [105]	2016	South korea	1371	49.3	13–15	Clinicalexamination and questionnaire	Erosion	High
Oginni A. O et al. [106]	2002	Nigeria	126	64.28	16	Clinicalexamination	Erosion	Moderate
Okawa Y et al. [107]	1993	Japan	770	3.2	20–59	Clinicalexamination	General	Moderate
Omar Abeer Bakr et al. [108]	2014	Saudi arabia	210	31.43	8–19	Questionnaire	Erosion	Moderate
Racki D. N. O et al. [109]	2020	Brazil	1197	57	15–19	Clinical examination and questionnaire	Erosion	High
Peres K et al. [110]	2020	Brazil	499	13	12	Clinicalexamination	Erosion	High
Pineda Aega et al. [46]	2019	Mexic	512	45.7	11–14	Clinicalexamination and questionnaire	Erosion	High
Prado I. M et al. [111]	2013	Brazil	172	81.6	12	Questionnaire	Attrition	Moderate
Provatenou E et al. [112]	2016	Greece	592	88.17	8–14	Questionnaire	Erosion	High
rios daniela et al. [113]	2007	Brazil	356	34.8	6	Clinicalexamination and questionnaire	General	Moderate
MabelMiluska Suca Salas et al. [114]	2017	Brazil	1210	25.1	8–12	Clinical examination and questionnaire	Erosion	High
Nilantha Ratnayake et al. [115]	2010	Srilanka	1200	22.4	17	Clinicalexamination and questionnaire	General	Moderate
Oliver Schierz et al. [116]	2014	Germany	836	23.4	20–59	Clinicalexamination	General	Moderate
Sandra Sagar et al. [117]	2020	India	258	21	12–80	Questionnaire	Erosion	High
D.A. Seligman et al. [118]	1988	USA	222	91.5	19–40	Clinicalexamination and questionnaire	General	Moderate
A Septalita et al. [119]	2017	Indonesia	487	88	12	Clinicalexamination	Erosion	High
Dr. Sahana Shivkumar et al. [120]	2011	India	210	52	5–10	Clinicalexamination and questionnaire	General	Moderate
DeeptiShrestha et al. [121]	2018	Nepal	364	60.1	15–75	Clinicalexamination and questionnaire	General	High
JENNYBOGSTAD SØVIK et al. [122]	2014	Norway	795	59	16–18	Clinicalexamination	Erosion	Moderate
Linastangvaltaite-Mouhat et al. [123]	2020	Lithuania	1397	38.3	35–74	Questionnaire	Erosion	High
B.G.N.smith et al. [124]	1996	UK	1007	87.9	6–14	Clinicalexamination	General	Moderate
Kan Sun et al. [65]	2017	China	1812	84.9	12–74	Clinical examination and questionnaire	General	High
Nayantara Sud et al. [125]	2015	India	100	13	20	Clinicalexamination and questionnaire	Abrasion	High
Izabela Strużycka et al. [126]	2014	Poland	1886	42.2	18	Clinicalexamination	Erosion	Moderate
Dan-Ying Tao et al. [127]	2015	China	1837	15.1	3–6	Clinical examination and questionnaire	Erosion	High
G.J. Truin et al. [128]	2005	The Netherlands	832	84.83	6–12	Clinical examination and questionnaire	Erosion	Moderate
MUHAMMAD WASEEM ULLAH KHAN et al. [129]	2018	Pakistan	2500	38.6	12–50	Clinicalexamination	General	High
UzmaShahbaz et al. [130]	2010	Pakistan	385	46	12–14	Clinicalexamination and questionnaire	Erosion	High
Nimet Ünlü et al. [131]	2014	Turkey	188	17.5	14–16	Questionnaire	Erosion	Moderate
A Wiegand et al. [132]	2006	Germany	463	32	2–7	Clinicalexamination	Erosion	Moderate
Fabiana VargasFerreira et al. [133]	2010	Brazil	944	7.2	11–14	Questionnaire	Erosion	High
Ping Wang et al. [134]	2010	China	1499	27.3	12–13	Clinical examination and questionnaire	Erosion	Moderate
Peter Wetselaar et al. [135]	2016	The netherland	1125	13	25–74	Clinicalexamination	General	Moderate
Zhao Wei et al. [4]	2014	China	720	64.8	35–74	Clinicalexamination	General	High
SangeetaYadav et al. [136]	2011	India	500	88	18–55	Clinicalexamination	Attrition	High
D. Williams et al. [137]	1999	UK	525	28.9	14	Clinical examination and questionnaire	Erosion	Moderate
Shinan Zhang et al. [138]	2014	Hong kong	704	21	12	Questionnaire	Erosion	Moderate
Andrius Zebrauskas et al. [139]	2014	Lithuania	132	35.6	12–25	Clinicalexamination and questionnaire	Erosion	High
Seong goo Yu et al. [140]	2013	South korea	788	27	9–10	Clinical examination and questionnaire	Erosion	Moderate

**Fig. 2:**
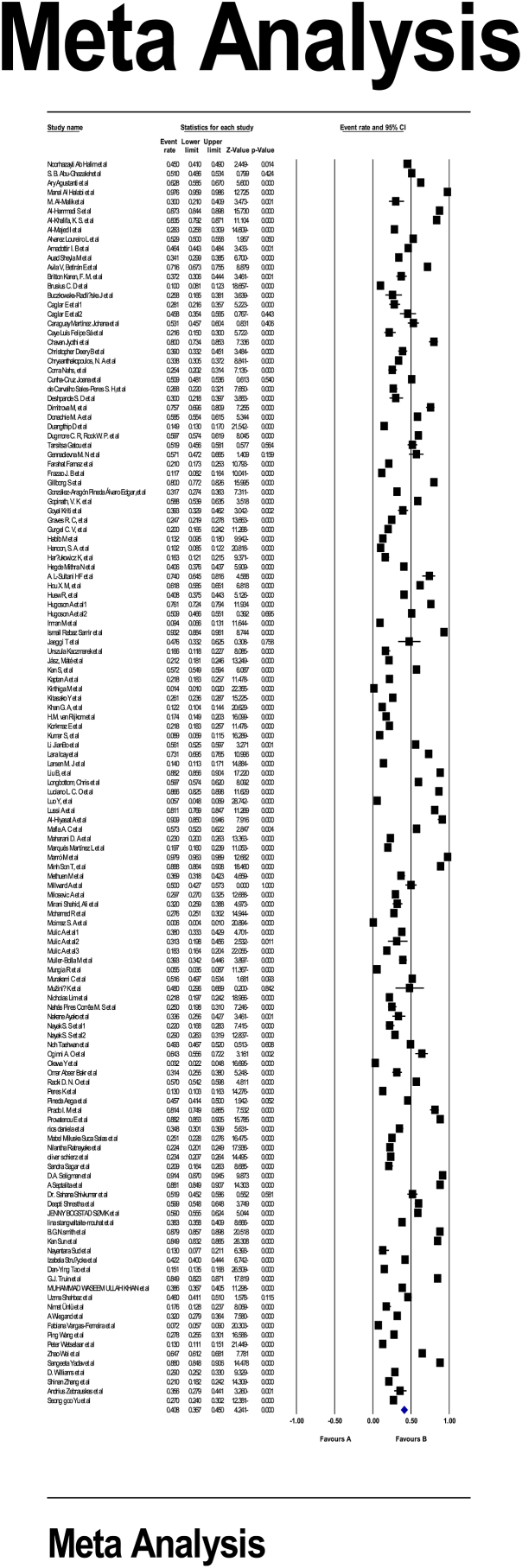
Forest plot of tooth wear prevalence based on the random effects method.

**Fig. 3:**
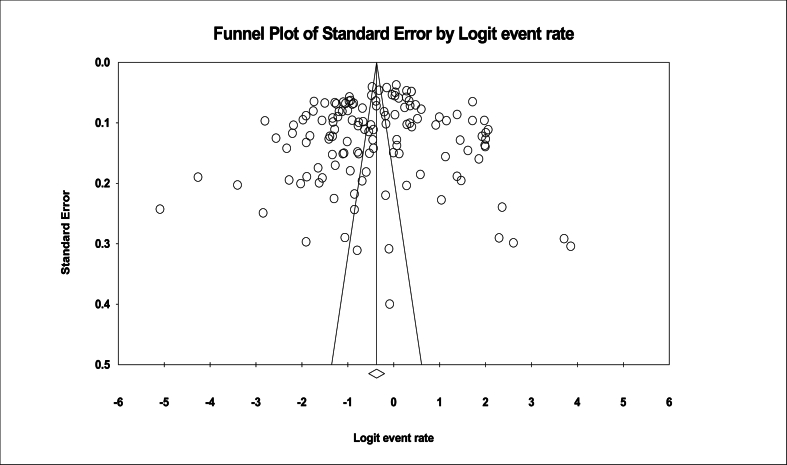
Funnel plot examining publication bias in reviewed studies.

In a review of 133 eligible studies with a sample size of 92,153 individuals, the I^2^ heterogeneity test was found at a high rate (I^2^:99.1); thus, the random effect model was used for meta-analysis. The global prevalence of TW was found 40.8 % (95 %CI: 36.7–45) (Fig. 2). Also, the Egger test showed no significant publication bias among the studies (p:0.346) (Fig. 3).

**Fig. 4:**
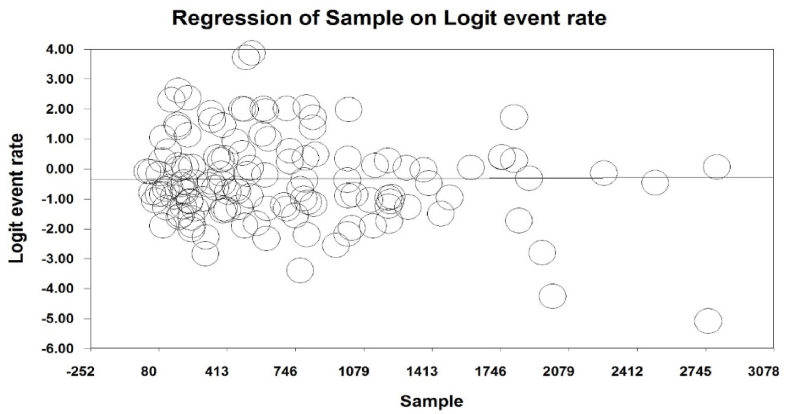
Meta-regression representing the effect of sample size on the prevalence of tooth wear.

**Fig. 5:**
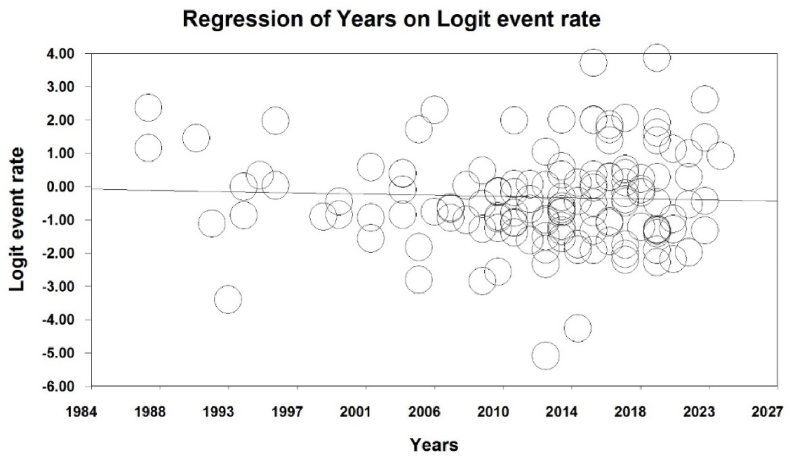
Meta-regression representing the effect of year of paper publication on the prevalence of tooth wear.

Meta-regression analysis revealed that following the increase in sample size (Fig. 4) and year of paper publication (Fig. 5), respectively, the prevalence of TW increases and decreases significantly (p < 0.05). Factors associated with TW are also listed in Table 2 based on the importance of the included studies (Table 2).

**Table 2:** Factors associated with tooth wear.

Factors	P-value
**GERD**	
Fabiana Vargas-Ferreira (2010) [1]	0.07
Tian Yu (2021) [2]	<0.001
Zhao Wei (2016) [4]	0.297
**Acid drinks**	
Fabiana Vargas-Ferreira (2010) [1]	0.81
Tian Yu (2021) [2]	0.003
Zhao Wei (2016) [4]	<0.001
**Male**	
Tatiana Pereira Cenci (2023) [8]	<0.001
Zhao Wei (2016) [4]	<0.001
Jenny Abanto (2014) [9]	0.798
**Female**	
Tatiana Pereira Cenci (2023) [8]	<0.001
Zhao Wei (2016) [4]	<0.001
Jenny Abanto (2014) [9]	0.798

## 4 Discussion

The present meta-analysis provided valuable insight into the global burden of TW, a multifactorial and progressive condition affecting the integrity and longevity of dental tissues. Despite being recognized as a common oral health concern, comprehensive data on the associated worldwide prevalence have been lacking, limiting the scope of preventive and restorative strategies in public health. The pooled results from 133 studies encompassing over ninety thousand participants revealed that 40.8 % of individuals in the general population exhibit some degree of TW. This striking figure emphasizes the widespread nature of the problem and highlights that attrition, erosion, abrasion, and abfraction collectively represent a major challenge to maintaining oral health. The high heterogeneity observed across studies suggests variations in diagnostic criteria, age distribution, and environmental factors, reinforcing the need for standardized methodologies and global cooperation to enhance surveillance and develop preventive protocols against TW.

The 99.1 % heterogeneity in prevalence studies is due to different populations and samples studied, different ages and sexes, different sample sizes and years of publication, different races, and different screening methods. We tried to investigate and report the impact of cases for which information was available through meta-regression based on the information obtained in the studies. According to this, with an increase in sample size, prevalence increased, and with an increase in the year of the study, prevalence decreased.

TW represents a progressive, multifactorial process characterized by the irreversible loss of enamel and dentin through mechanical and chemical mechanisms such as attrition, abrasion, erosion, and abfraction. TW represents various mechanisms, including friction with other teeth and foreign bodies or dissolution in liquids (especially acids). Thus, the diversity of mechanisms causes different types of TW [141]. It is considered a growing public health concern because it not only compromises dental structure but also affects functional, esthetic, and psychological aspects of oral health [142]. As enamel thins and dentin becomes exposed, individuals experience increased tooth sensitivity, a heightened risk of dental caries, and potential pulpal inflammation. These structural changes can impair mastication, alter occlusal relationships, and contribute to temporomandibular discomfort and muscle fatigue [143]. Moreover, visible shortening and discoloration of teeth may reduce self-esteem and social confidence, highlighting TW's broader impact on oral-health-related quality of life [144]. The increasing prevalence of TW among younger populations underscores its clinical relevance, linking it not only to dietary acid consumption and GERD but also to lifestyle and behavioral factors. Understanding its biological mechanisms and clinical importance enables clinicians to develop preventive protocols and patient-centered restorative approaches that preserve function, aesthetics, and overall well-being.

Erosive TW has emerged as a prevalent and complex dental condition with substantial public health relevance, as highlighted in recent systematic reviews and meta-analyses. Dallavilla et al. (2024) synthesized data from numerous high-risk populations, revealing that erosive TW prevalence varies profoundly depending on patient background: 30 % among medication users, 67 % among those exposed to drugs and alcohol, 54 % among individuals with chronic GERD, and as high as 65 % in groups with eating disorders or restrictive/special diets [145]. Importantly, these studies demonstrate that the interplay of lifestyle, medical conditions, and behavioral factors can dramatically increase vulnerability to erosive tooth damage within specific demographics. The study also emphasizes the need for clinicians to routinely assess patients' medical and dietary history to identify those at elevated risk and implement targeted preventive and therapeutic interventions. The burden of erosive TW in children and young populations is equally apparent. In the 2025 meta-analysis, Marschner et al. reported an overall prevalence of 35.6 % (95 % CI: 24.8–48.1) in children with primary dentition. Notably, the risk nearly doubled in children with GERD (OR = 1.98, 95 % CI: 1.37–2.87), and was significantly higher among those who reported regular consumption of acidic foods (OR = 5.14, 95 % CI: 3.56–7.42) and acidic drinks (OR = 6.90, 95 % CI: 4.64–10.25) [146]. These findings, echoed by Yip et al. (2022), indicate that approximately 39.6 % (95 % CI: 27.6–51.6) of preschool-aged children show evidence of ETW, confirming the early onset of the disease. Given the potential for lifelong impact, early detection and education around dietary habits, particularly regarding acid exposure, have become central to pediatric dental protocols [147]. According to a study of Perez et al. (2008) on 12-year-old children, the prevalence of TW was found 26.9 %, while this value was reported 63 % by Agustanti et al. (2018) [5,35]. According to the studies conducted by Hegde et al. (2018) and Hugh et al. (2012), the prevalence of TW was found 40.6 % and 40.8 %, respectively [54,57], which was relatively similar to our findings. Maro et al. found that 97.9 % of individuals are diagnosed with TW. This high rate finding was probably associated with the high prevalence of risk factors in the studied samples, along with social, economic, cultural, and health differences [85].

Regional epidemiological investigations present an even more granular view. Yu et al. (2021) documented a pronounced prevalence gradient in Shanghai, where 59.7 % of adolescents and 93.1 % of adults were affected by TW. Severity correlated directly with age, GERD, dry mouth, and frequent soft or alcoholic drink intake. These data reinforce the dynamic nature of risk over the lifespan, highlighting that both biological and environmental factors—such as reduced salivary flow—amplify susceptibility in older populations [148]. In Southeast Asia, Lim et al. (2022) observed a 32.8 % prevalence among Singaporean military young adults, with significant roles attributed to habitual carbonated beverage intake and parafunctional behaviors (e.g., bruxism, aggressive brushing) [149]. The pan-European investigation by West et al. (2024) expanded upon these findings, revealing that more than one-third of adult participants from seven countries presented with clinically evident TW. This was frequently accompanied by other oral conditions, including dentine hypersensitivity and gingival recession, suggesting shared risk factors and potentially synergistic pathological processes [150]. These findings further support the multifactorial pathway of ETW, where both intrinsic acids (from gastric reflux) and extrinsic acids (from diet) create an oral environment hostile to tooth enamel. The acids dissolve hydroxyapatite crystals, undermining the mineral structure, while mechanical abrasion, such as toothbrushing with abrasive pastes or bruxism, exacerbates enamel and dentin loss.

In terms of prevention and management, current research points to the value of individualized strategies, including dietary counseling, medical management of GERD, and the use of remineralization therapies such as fluoride varnishes and novel bioactive coatings. The adoption of standardized diagnostic indices, like the Basic Erosive Wear Examination (BEWE), allows for consistent assessment and international comparison, while also guiding intervention thresholds [151].

Collectively, these landmark studies illustrate the alarming and global nature of ETW across age groups and populations. The progressive, irreversible character of the disease, coupled with its diverse etiological pathways—ranging from systemic conditions to everyday habits—mandates a multidisciplinary approach for effective prevention, early detection, and minimally invasive restorative care. Proactive strategies, patient education, and regular screening are crucial for mitigating long-term impacts and preserving oral health into older age [152].

Patients with tooth erosion may complain of tooth sensitivity, functional impairment due to occlusal changes, and deterioration of esthetic appearance due to discoloration or shortening of teeth. While this condition can have a wide impact on the clinical aspects and consequences of tooth erosion, it also has implications for patients' oral health, daily functioning, and quality of life [152]. In a study by Mireille Kanaan et al., it was reported that although the prevalence of tooth erosion was high, its severity and impact on oral health-related quality of life in adults with complete dentition or with several missing posterior teeth was limited. Indicators such as age, lack of access to regular oral health care due to the cost of care, tooth sensitivity, changes in tooth appearance, and daily consumption of acidic beverages were mediators of impaired oral health-related quality of life [152]. While Mehta et al., in their study in the UK, Malta, and Australia, reported that higher levels of tooth wear were significantly associated with reduced oral health-related quality of life among participants [153].

One of the main limitations of the present study is the small number of eligible investigations and the presence of many non-English investigations with lost data. In addition, TW is a challenge which is caused by various factors, including lifestyle, which was ignored in some studies. The lack of access to the full text of some studies, along with unextractable data, also created a serious limitation in this regard.

### 4.1 Strengths and limitations

The most important strengths of this study were the comprehensive review of all databases and information sources, as well as the presentation of the overall prevalence and the examination of heterogeneity factors through meta-regression, which can improve future studies in this field. Also, the presentation of this prevalence provides a way to provide intervention programs, and the impact of intervention in reducing prevalence in the population can be compared.

### 4.2 Conclusion

According to the results of this study, the global prevalence of TW is 40.8 % with an increasing rate annually. Thus, this finding is useful for health policymakers to design a comprehensive treatment strategy to prevent TW in individuals at risk.

## Ethical statement

Ethics approval was received from the ethics committee of deputy of research and technology, Kermanshah University of Medical Sciences (50005877).

## Availability of data and materials

Datasets are available through the corresponding author upon reasonable request.

## Authors'contributions

NS and AHS and MM contributed to the design, MM statistical analysis, and participated in most of the study steps. MM and AA and HZ prepared the manuscript. MM and AHG and SHSH assisted in designing the study, and helped in the, interpretation of the study. All authors have read and approved the content of the manuscript.

## Funding

By Deputy for Research and Technology, Kermanshah University of Medical Sciences (IR) (50005877). This deputy has no role in the study process.

## Declaration of competing interest

The authors declare that they have no known competing financial interests or personal relationships that could have appeared to influence the work reported in this paper.
